# Possible spread of SARS-CoV-2 in domestic and wild animals and body temperature role

**DOI:** 10.1016/j.virusres.2023.199066

**Published:** 2023-02-10

**Authors:** Zinaida Klestova

**Affiliations:** Institute for Medical Virology and Epidemiology of Viral Diseases, University Hospital Tübingen, Elfriede-Aulhorn-Straße 6, Tübingen 72076, Germany

**Keywords:** SARS-CoV-2, Reservoirs, Animals, Regional spreading, Body temperature role

## Abstract

The COVID-19 pandemic was officially announced in March 2020 and is still moving around the world. Virus strains, their pathogenicity and infectivity are changing, but the ability is fast to spread and harm people's health remained, despite the seasonality seasons and other circumstances. Most likely, humanity is doomed for a long time to coexistence with this emergent pathogen, since it is already circulating not only among the human population, but and among fauna, especially among wild animals in different regions of the planet. Thus, the range the virus has expanded, the material and conditions for its evolution are more than enough.

The detection of SARS-CoV-2 in known infected fauna species is analyzed and possible spread and ongoing circulation of the virus in domestic and wild animals are discussed. One of the main focus of the article is the role of animal body temperature, its fluctuations and the presence of entry receptors in the susceptibility of different animal species to SARS-CoV-2 infection and virus spreading in possible new ecological niches. The possibility of long-term circulation of the pathogen among susceptible organisms is discussed.

## Highlights

• New ecological niche for SARS-CoV-2.
• Circulation of the SARS-CoV-2 among different animal species.
• Reasons allowing the SARS-CoV-2 to find new ecological niches.
• Danger to humans of prolonged circulation of the SARS-CoV-2 in animals.

## Introduction

„As of 30 April 2022, around 500 million human cases of SARS-CoV-2 infection have been confirmed worldwide, with more than 6 million human deaths from COVID-19. The nature of this new virus, together with its widespread distribution and the susceptibility of some animal species to infection, manifests in animal infections arising from close contact between humans and animals. Conversely, there is also evidence that, for some animal species, close contact with infected animals can represent a potential source of infection in humans” (SARS-COV-2 in animals – situation report 12 30/04/2022; Murphy and Ly, 2021; Tiwari et al., 2020). Recently 623,951,852 confirmed human cases of COVID-19 have been reported worldwide, with 6,735,439 human deaths (on 20.01.2023, https://coronavirus.jhu.edu/. COVID-19 has had a serious negative impact on the human population worldwide, but so far little is known about how the SARS-CoV-2 virus affects wild and domestic animals.

A few important questions remain unanswered: What are the natural reservoirs of SARS-CoV-2? Which species transmitted the virus to humans? In which animals can the virus circulate in nature?

Since the initial outbreak of COVID-19 in 2019, there have been numerous reports of SARS-CoV-2 infection of animals (SARS-COV-2 in animals – situation report 12 30/04/2022; SARS-COV-2 in animals – situation report 12 30/04/2022 2022, SARS-COV-2 in animals – situation report 1 31/05/2021). There is also evidence that, for some animal species, close contact with infected animals may represent a potential source of infection for humans. To date, all cases of SARS-CoV-2 described in animals have been linked to reverse zoonosis (human to animal transmission) from zoo or mink farm workers or owners living with dogs and cats (Drozdz et al., 2021; Decaro et al., 2021; Miro et al., 2021). In this article not only the "One Health Concept" had been implemented, but also had been discussed possible reservoir species of the virus in nature.

The idea of the analysis presented in this paper was to identify: - the distribution range of SARS-CoV-2 among animals in different regions of the planet; - some conditions conducive to the long-term persistence of infectious properties of the virus (temperature regimes) and its subsequent transmission to susceptible organisms;-data on the newly animal coronaviruses and discussion on the possibilities of interspecific transmission;-the causes of carriage and transmission of SARS-CoV-2 in animals, presumably related to their body temperature and the presence of a specific receptor for the virus.

These issues are closely related and are therefore analyzed in more detail in the sections below.

## 1 Analysis of animal reservoirs of SARS-CoV-2

### 1.1 General problems of virus transmission in animal/human

The exact animal reservoir of SARS-CoV-2 is still unknown despite reports of SARS-CoV-2-related viruses in Asian bats and pangolins (SARS-COV-2 in animals – situation report 1 31/05/2021; Temmam et al., 2022; Zhou et al., 2020; Zhou et al., 2021; Wacharapluesadee et al., 2021; SARS-COV-2 in animals – situation report 1 31/05/2021 2021). SARS-CoV-2 is thought to have originated in bats; however, the intermediate animal sources of this virus are unknown (Shi et al., 2020). Much remains to be learned about how SARS-CoV-2 affects different animal species. The first case of SARS-CoV-2 in pets was officially reported to WOAH (former OIE) (SARS-COV-2 in animals – situation report 1 31/05/2021 2021), changing the perception that SARS-CoV-2 cannot transmit thoughts to domestic animals such as cats and dogs. In first report of OIE (published on 31/05/21) reported about total 101 cats and 85 dogs number of outbreaks worldwide (SARS-COV-2 in animals – situation report 1 31/05/2021 2021). A 17-year-old neutered male Pomeranian, is the first dog tested positive for SARS-CoV-2, with a history of multiple medical conditions including grade II heart murmurs, systemic and pulmonary hypertension, chronic kidney disease, hypothyroidism, and hyperadrenocorticism. The owner of this dog was a 60-year-old woman who developed symptoms on February 12, 2020 and was diagnosed with COVID-19 on February 24, 2020. These were the first confirmed cases of transmission of infection from humans to animals. The second dog with this virus was a 2.5-year-old German Shepherd in good health from the household whose owner developed symptoms on March 10, 2020, and was diagnosed with COVID-19 on March 17, 2020 (Miro et al., 2021). Later the virus was also detected in dogs in Uruguay (SARS-COV-2 in animals – situation report 1 31/05/2021; SARS-COV-2 in animals – situation report 1 31/05/2021 2021). To date, have been reported sporadic cases of SARS-CoV-2 infection in dogs and cats (Decaro et al., 2021). There is evidence that it is possible for SARS-CoV-2 transmission from humans to animals. Infected dogs had any specific symptoms while they were excreted with the virus (Sit et al., 2020).

In March 2020 the first case occurred SARS-CoV-2 in a cat in Brussels whose owner had just returned from a vacation in Italy (Garigliany et al., 2020). It is thought that pets can become infected from humans. However, cats and dogs are not easily infected in vivo, and there was no evidence before that infected cats or dogs spread the virus to other animals or humans. There is evidence to suggest that dogs have low susceptibility to SARS-CoV-2 (Murphy and Ly, 2021; Schoemaker, 2008). In the first wave of the COVID-19 pandemic, 2257 oropharyngeal and nasal swab samples from 877 dogs and 260 cats (including 18 animals from COVID-19-affected households and 92 animals with signs of respiratory disease) were analyzed. These studies led to the hypothesis that the low prevalence of SARS-CoV-2 infection among domestic cats and dogs and, therefore, the risk of zoonotic transmission to veterinary personnel was low during the first wave of the pandemic, even in hotspots (Klaus et al., 2021). But, in experimental conditions, it has been proven that an infected cat can transmit the virus to other susceptible cats living with it (Shi et al., 2020). Infected cats can show any or light signs of illness (Mallapaty, 2020). A tiger from the Bronx Zoo that tested positive for SARS-CoV-2 contracted mild respiratory disease (Tiwari et al., 2020). He demonstrated that cats can spread the virus by airborne droplets (Shi et al., 2020). In animals, the presence of the SARS-CoV-2 is confirmed by the following methods: virus isolation, RT-PCR, PT-LAMP, ELISA antibody test, virus genome sequencing, virus neutralization test (OIE 2020).

SARS-CoV-2 has also been diagnosed in non-domestic animal species, but evidence suggests that these infected animals also pose little risk to humans - even to those who work closely with them, as long as appropriate protective equipment is used. Although there is considerable debate about the importance of bats (and pangolins) as carriers of viruses that could one day spread to humans (Temmam et al., 2022; Zhou et al., 2020; Wacharapluesadee et al., 2021). Some authors believe that it is too early to confirm the role of intermediate hosts such as snakes, pangolins, turtles and other wild animals in the origin of SARS-CoV-2, in addition to bats, natural hosts of numerous coronaviruses such as SARS-CoV and MERS-CoV (Tiwari et al., 2020). As interspecific transmission was an open question, it stimulated an active search for infected animals (both wild and domestic).

#### 1.1.1 SARS-CoV-2 infected animals or possible transmitters

However, recent animal studies have identified a wide range of animals susceptible to and/or infected with SARS-CoV-2 worldwide. Infections have been reported in 23 animal species from 36 countries in the Americas, Africa, Asia and Europe (SARS-COV-2 in animals – situation report 12 30/04/2022; SARS-COV-2 in animals – situation report 1 31/05/2021 2021, OIE, 2022). These include domestic cats *(Felis catus)* (Decaro et al., 2021; Miro et al., 2021; Garigliany et al., 2020; Klaus et al., 2021; Jairak et al., 2022) and domestic dogs *(Canis lupus familiaris)* (Decaro et al., 2021; Miro et al., 2021; Shi et al., 2020; Sit et al., 2020; Klaus et al., 2021; Jairak et al., 2022; Bosco-Lauth et al., 2020; Patterson et al., 2020; de Morais et al., 2020), minks *familie: Mustelinai, (Neovison vison or “American mink”, Mustela lutreola, or “ European mink, the “Russian mink”, or “Eurasian mink”))* (Boklund et al., 2021; Shriner et al., 2021; Oreshkova et al., 2020), otters *(familie Mustelidae)* (SARS-COV-2 in animals – situation report 12 30/04/2022, SARS-COV-2 in animals – situation report 1 31/05/2021; Confirmation of COVID-19 in Otters at an Aquarium in Georgia 2021), pet ferrets *(Mustela putorius furo)* (SARS-COV-2 in animals – situation report 12 30/04/2022; Shi et al., 2020; van den Brand et al., 2008; Alluwaimi et al., 2020; Zhang et al., 2020a), lions *(Familie: Felidae*, Panthera leo) (SARS-COV-2 in animals – situation report 12 30/04/2022, SARS-COV-2 in animals – situation report 1 31/05/2021; Mishra et al., 2021; Koeppel et al., 2022), tigers *(Familie: Felidae, Panthera tigris)* (SARS-COV-2 in animals – situation report 12 30/04/2022, SARS-COV-2 in animals – situation report 1 31/05/2021; Mitchell et al., 2021; Grome et al., 2022), pumas (cougar) *(Felis concolor)* (SARS-COV-2 in animals – situation report 12 30/04/2022, SARS-COV-2 in animals – situation report 1 31/05/2021; Koeppel et al., 2022), snow leopards *(Panthera uncia****)*** (SARS-COV-2 in animals – situation report 12 30/04/2022, SARS-COV-2 in animals – situation report 1 31/05/2021; Wang et al., 2022), gorillas *(Gorilla gorilla)* (SARS-COV-2 in animals – situation report 12 30/04/2022, SARS-COV-2 in animals – situation report 1 31/05/2021; Erste Gorillas mit dem Coronavirus infiziert, in Scinexx 2022), white-tailed deer *(Odocoileus virginianus)* (Chandler et al., 2021; Questions and Answers: Results of Study on SARS-CoV-2 in White-Tailed Deer 2021), fishing cats *(Prionailurus viverrinus)* (SARS-COV-2 in animals – situation report 12 30/04/2022, SARS-COV-2 in animals – situation report 1 31/05/2021; Confirmation of COVID-19 in a Binturong and a Fishing Cat at an Illinois Zoo 2021), Binturongs (*Arctictis binturong*) (SARS-COV-2 in animals – situation report 12 30/04/2022; Confirmation of COVID-19 in a Binturong and a Fishing Cat at an Illinois Zoo 2021), South American coati *(Nasua nasua)* (SARS-COV-2 in animals – situation report 12 30/04/2022; Confirmation of COVID-19 in a Coatimundi at an Illinois Zoo 2021), spotted hyenas *(Crocuta crocuta*) (SARS-COV-2 in animals – situation report 12 30/04/2022; East et al., 2004), manatees *(genus Trichechu*s) (SARS-COV-2 in animals – situation report 12 30/04/2022), Eurasian lynx *(Lynx lynx)* [(SARS-COV-2 in animals – situation report 12 30/04/2022, SARS-COV-2 in animals – situation report 1 31/05/2021),], Canadian lynx *(Lynx canadensis*) (SARS-COV-2 in animals – situation report 12 30/04/2022, SARS-COV-2 in animals – situation report 1 31/05/2021; Lynx TESTs POSITIVE FOR SARS-CoV-2 2021), hippopotamus *(Hippopotamus amphibius)* (SARS-COV-2 in animals – situation report 12 30/04/2022; Hayashi, 2022), hamsters *(Family: Cricetidae, subfamily Cricetinae)* (SARS-COV-2 in animals – situation report 12 30/04/2022; Bosco-Lauth et al., 2020; Alluwaimi et al., 2020), mule deers *(Odocoileus hemionus)* (SARS-COV-2 in animals – situation report 12 30/04/2022; Alluwaimi et al., 2020; Hale et al., 2022), skunks *(family Mephitidae)* (Bosco-Lauth et al., 2021), giant anteaters *(Myrmecophaga tridactyla)* (SARS-COV-2 in animals – situation report 12 30/04/2022; Castagnino, 2021), West Indian manatees *(Trichechus manatus)* (SARS-COV-2 in animals – situation report 12 30/04/2022), black-tailed marmosets *(Mico melanurus)* (SARS-COV-2 in animals – situation report 12 30/04/2022; Pereira et al., 2022). An updated list of animal species infected with SASR-CoV-2 in US can be accessed via the link of the US Department of Agriculture (SARS-COV-2 in animals – situation report 12 30/04/2022 2022).

While human-to-human transmission is the main driver of the pandemic in communities and internationally, the number of animal infections of SARS-CoV-2, although still isolated, continues to increase. There are currently 675 animal outbreaks worldwide on 30/04/2022 and 676 on 31/05/2022. (SARS-COV-2 in animals – situation report 12 30/04/2022, OIE, 2022).

According to the WOAH report of 30/04/2022 most of the animal species, in which cases of SARS-CoV-2 have been detected by different regions of the world are in the Americas region (20 species in total): cat, dog, mink, otter, pet ferret, lion, tiger, puma, snow leopard, gorilla, white-tailed deer, fishing cat, binturong, coatimundi, spotted hyena, Canada lynx, mule deer, giant anteater, West Indian manatee, black-tailed marmoset. In contrast, less infected species were reporter from Africa 2 species (lion and puma), Asia (5 species: cat, dog, lion, tiger, hamster) and Europe (8 species: cat, dog, mink, pet ferret, lion, tiger, Eurasian lynx, hippopotamus) (SARS-COV-2 in animals – situation report 12 30/04/2022). One month later, another outbreak was added in 1 additional European country (Switzerland) (OIE, 2022).

Comparing the analysis of detected SARS-CoV-2 animal cases based on WOAH reports for 2022 (SARS-COV-2 in animals – situation report 12 30/04/2022) with the situation one year earlier (on 31/05/2021) (SARS-COV-2 in animals – situation report 1 31/05/2021) it can be noted that in 2021, the majority of cases in different regions of the planet occurred in the Americas region, but compared to 2022, only 9 animal species were known to be infected in 2021. These findings are surprising because COVID-19 started from the Asian region. This suggests that the virus has either spread further and crossed the species barrier to new species and/or that surveillance and monitoring of animals has become better and more widespread. In Africa, only one animal species (cougar) was reported infected in 2021 and another (lion) in 2022. Thus, based on published data, Africa has the least information on animals infected with SARS-CoV-2. There is no information from the Australian region. In 2021, only two animal species (cat and dog) were infected with SARS-CoV-2 in Asia. But in 2022, more animals were recorded in Asia infected with SARS-CoV-2: lion, tiger and hamster. In Europe, six species of infected animals were reported in 2021 and two more species were added in 2022. It is interesting that the data given for a one-month period shows the extent to which the number of infected animals has increased. For example, during one month (01/05/2021 - 31/05/2021), there were 13 outbreaks of SARS-CoV-2 in 4 animal species (cat, dog, mink and tiger) in 5 countries (SARS-COV-2 in animals – situation report 1 31/05/2021). This worries us because there is indeed a suspended possibility of rapid evolution of the virus and the emergence of new variants. This danger is confirmed by the detection of the virus in primates. For example, the virus was first detected in gorillas in January 2021 from the San Diego Zoo Safari Park (Erste Gorillas mit dem Coronavirus infiziert, in Scinexx 2022). These are the first documented cases of COVID-19 in great apes worldwide. Some evidence also suggests that free-ranging non-human primates are potential carriers of natural SARS-CoV-2 infection (Pereira et al., 2022) as non-human primates infected with SARS-CoV-2 may show mild clinical signs (Munster et al., 2020; Rodriguez-Morales et al., 2020). Biologists have long feared that the pandemic could threaten populations of great apes in Africa and Asia, which are already at risk.

The SARS-CoV-2 cell entry receptor, as in humans, is ACE2 also in dogs, cats, tigers, hippos and many other animal species (Hayashi, 2022). Significant studies have been performed on a variety of domestic and laboratory animals and birds to identify the clinical manifestations of SARS-CoV and SARS-CoV-2 (Alluwaimi et al., 2020). Ferrets and cats have been found to be highly susceptible to SARS-CoV-2. These two species differ only in two amino acids in the SARS-CoV-2 spike-contacting regions of their ACE2 orthologues (Shi et al., 2020). It has been reported that cats in Wuhan were seropositive to SARS-CoV-2 during and after the COVID-19 outbreak. No cross-reactivity was found between SARS-CoV-2 and other feline enteric coronaviruses (FeCV) type I or II, which cause feline infectious peritonitis (FIP) (Zhang et al., 2020a). Surveillance of SARS-CoV-2 in cats should be considered in addition to the prevention of COVID-19 in humans (Shi et al., 2020).

So far, WHO has declared the unclear of origin of SARS-CoV-2. In connection with further research (P. Zhou et al., 2020; Pizzato et al., 2022), it was hypothesized that the site of animal-to-human transmission was the wet markets of Wuhan, which was not confirmed (Malaiyan et al., 2021) as animal samples collected in the marketplace did not allow accurate identification of the zoonotic precursor strain. Interestingly for us, one of the closest virus strains to SARS-CoV-2, RaTG13, with 96.2% (P. Zhou et al., 2020) genomic sequence identity, was found in a horseshoe bat (*Rhinolophus affinis* from Yunnan province, China). The same viral splicing protein site was found in a strain of coronavirus derived from the Malayan pangolin (pangolin-CoV-2020), so it was hypothesized that this animal could be an intermediate host for SARS-CoV-2. However, careful research has not confirmed this (Malaiyan et al., 2021). Pangolin virus and other SARS-CoV-2-like viruses have been detected in bats in various locations in South-East Asia. 293 different coronaviruses were found out of 1322 samples from bats - 284 were alpha-coronaviruses and 9 were beta-coronaviruses (SARS-CoVs). In 2020, SARS-CoV-2 sequences were compared with RdRp sequences of bat coronaviruses. They found identity with RaTG13 (96.2%) (Zhou et al., 2020c). SARS-CoV-2 was found to be 88–89% identical to two other bat SARS coronaviruses (bat-SL-CoVZC45 and bat-SL-CoVZXC21, also named ZC45 and ZXC21) (Malik et al., 2020).In laboratory studies, other authors have confirmed that SARS-CoV-2 is 96% identical to bat CoV at the genomic level and believe that bats may be the main source of this zoonotic spread of the virus. (Tiwari et al., 2020; Rodriguez-Morales et al., 2020; Malik et al., 2020). Importantly, two highly significant endogenous pre-pandemic SARS-like bat coronaviruses (WIV1-CoV and SHC014-CoV) can replicate relatively efficiently in an experiment based on specific mice (LoMs). Virus replication in this model occurs in lung tissue and does not require any adaptation by the pathogen or the host. Coronaviruses circulating in bats have been found to have pandemic potential without the need for adaptation to humans (Wahl et al., 2020).

SARS-CoV-2 is not identical although phylogenetically similar to pangolin CoV-2020 coronaviruses, RaTG13 and another bat CoV. Although bats are likely reservoirs for this virus, it is likely that other mammalian species may be intermediate hosts. In these unknown intermediate hosts, SARS-CoV-2 may have acquired some or all of the mutations necessary for effective transmission and replication in humans (Pizzato et al., 2022; Zhang and Holmes, 2020). Since at the beginning of the pandemic it was thought that pangolins and bats were possible reservoirs of the virus, consider these assumptions.

##### Investigation on SARS-CoV-2 in pangolins

Early in the COVID-19 pandemic, it was reported that viruses related to SARS-CoV-2 were detected in Asian *Rhinolophus* bats and in pangolins (Temmam et al., 2022; Shi et al., 2020; Liu et al., 2020; Xiao et al., 2021; Wahba et al., 2020; Delaune et al., 2021). It has been suggested that pangolins were potential reservoirs of SARS-CoV-2. But, opinion that pangolins may serve as intermediate hosts for SARS-CoV-2 are highly controversial (Tiwari et al., 2020). However, in 2020, the authors thought that further studies were needed to clarify whether SARS-CoV-2 S protein binds to pangolin ACE2 (Damas et al., 2020). This response followed later published data from other authors (Wrobel et al., 2021; Zhang et al., 2021; Niu et al., 2021) who showed that S protein from Guangdong pangolin-CoVs, a closely related SARS-CoV-2, bind strongly to human and pangolin ACE2 receptors. They also showed that the spike of pangolin-CoVs adopts a completely closed conformation and, more similar to that of bat coronavirus RaTG13 than to SARS-CoV-2 (Wrobel et al., 2021). However, other authors found that the pangolin-CoV GX spike, but not RaTG13, is comparable to the SARS-CoV-2 spike in binding the human ACE2 receptor and supporting pseudo virus entry into cells. Critical residues in RBD underlying the different activities of RaTG13 and pangolin-CoV GX/SARS-CoV-2 spikes were identified. Taken together, these results show that tight RBD-ACE2 binding and efficient of receptor-binding protein spike domain (RBD) conformational sampling are essential for the evolution of SARS-CoV-2 to achieve highly efficient infection (Zhang et al., 2021). RBD of pangolin-CoVs was found to bind to hACE2 as efficiently as the RBD of SARS-CoV-2 in vitro (Niu et al., 2021). The introduction of a Q498H substitution in RBD SARS-CoV-2 was found to extend the binding ability to mouse, rat and European hedgehog ACE2 homologues. These data suggest that these two pangolin-CoVs can infect humans, highlighting the need for further surveillance of pangolin-CoVs (Niu et al., 2021).

Consider the situation where pangolins, or bats (or other animals) from which Chinese scientists have isolated the SARS-Cov-2 virus and which prove identical to the human coronavirus in the current pandemic, may be possible carriers of the virus in the wild and try to analyses such a situation. We do not consider various conspiracy theories of the virus's emergence. Let us assume a natural origin of SARS-CoV-2. Firstly, needs to be analyzed whether it is possible that pangolins are a species that could be a reservoir of the virus.

We consider information from the published literature that pangolins belong to the mammalian or pangolinic family (2*n* = 42). Fossil remains are known from the Early and Middle Paleogene of North America, the Middle Paleogene and Early Neogene of Europe, and from the Early Paleogene pangolins are known from Africa and South Asia. Although the exact evolutionary origin of pangolins is unclear, they are known to belong to an ancient group of mammals. They are thought to have evolved around 55 million years ago. They are equipped with ‘armour’ made of scales, hence the animal's name, ‘shell’ (the skin of these animals). Pangolins are consumed as food in some Asian countries and their scales are used in traditional medicine. Although trapping is prohibited in China, pangolins are actively being destroyed and are illegally traded.

Habitat destruction and deforestation have reduced pangolin numbers. According to ZSL London Zoo (https://www.zsl.org/species-of-pangolins), USAID (https://www.usaidrdw.org/resources/pangolin-species-identification-guide/pangolin-id-guide-rast-english.pdf) and African Wildlife Foundation (https://www.awf.org/wildlife-conservation/pangolin) eight species of pangolins are found on two continents. Four species live in Africa: Black-bellied pangolin *(Manis (Phataginus or Uromanis) tetradactyla),* White-bellied pangolin (*Manis (Phataginus) tricuspis)*, Giant Ground pangolin (*Manis (Smutsia) gigantea*) and Temminck's Ground pangolin (*Manis (Smutsia) temminckii*). The four species found in Asia: Indian pangolin (*Manis crassicaudata*), Philippine pangolin *(Manis culionensis*), Sunda pangolin (*Manis javanica*) and the Chinese pangolin (*Manis pentadactyla).*

##### Search for SARS-CoV-2 in bats and newly discovered animal coronaviruses

The more attention is paid to coronaviruses, the newer species are found. New and new information about the appearance of new members of this family is added to the previously discovered and identified coronaviruses. As for examples, newly discovered Alphacoronaviruses (last 10–17 years) include the following species: six bat coronaviruses, namely: CDPHE15; HKU10; Mimiopterus bat coronavirus 1; Mimiopterus bat coronavirus HKU8; Rhinolophus bat coronavirus HKU2; Scotophilus bat coronavirus 512.

Notably, three more Betacoronaviruses have recently been detected in bats: Pipistrellus bat coronavirus HKU5; Rousettus bat coronavirus HKU9; Tylonycteris bat coronavirus HKU4. Furthermore, Deltacoronaviruses have been newly discovered in the following bird species: common chicken (Common moorhen coronavirus HKU21), night heron (Night heron coronavirus HKU19), sparrow (HKU 17), magpie (HKU18), small bird Japanese white-eye (white-eye COV HKU16); wigeon (Wigeon CoV HKU20), thrush (thrush CoV HKU12), and bulbul (BuCoV HKU11 or Bulbul coronavirus HK U11) (Woo et al., 2012).

Coronavirus infection (caused by coronavirus antigenic group 1) in hyenas (Crocuta crocuta) was first reported in 2004. Evaluation of phylogenetic relationships between coronavirus isolates (non-SARS-CoV-2) showed significant divergence between the hyena coronavirus variant and coronavirus variants of European, American and Japanese domestic cats and dogs. Sequencing of two amplified products from the protein S gene of the positive sample revealed the presence of specific coronavirus RNA with sequence homology with canine coronavirus 76 and 78% and feline coronavirus type II 80 and 84%, respectively (East et al., 2004). During long-term observations of several hundred known individuals, the only clinical sign in hyenas consistent with that described for coronavirus infections in dogs and cats was diarrhea. There was no evidence that coronavirus infection in hyenas causes clinical signs similar to feline infectious peritonitis in domestic cats or is a direct cause of mortality in hyenas. High susceptibility to coronavirus and excretion of coronavirus were first demonstrated in free-ranging spotted hyenas (East et al., 2004). However, there are now known cases where SARS-CoV-2 has been detected in spotted hyenas (SARS-COV-2 in animals – situation report 12 30/04/2022; East et al., 2004).

We have provided these examples to illustrate that our knowledge of emerging pathogens and their respective habitats is expanding every year.

The diversification of CoVs in terms of their inter- and intraspecific transmission is closely linked to the genome structure, replication and transcription mechanisms of CoVs (Ye et al., 2020). Mutation rates in the RNA of CoVs are estimated to be moderate to high. The average substitution rate for CoVs was ∼10^−4^ substitutions per year per site (Su et al., 2016).

In a US study, antibodies to SARS-CoV-2 were detected in only 1 of 143 white-tailed deer blood samples collected before January 2020 (before the human COVID-19 pandemic) (Zhang et al., 2020a). But between January 2020 and March 2021, 33% of 481 samples were positive for antibodies to SARS-CoV-2 (Zhang et al., 2020a). It remains unclear whether the reindeer got the virus from humans, from the environment or from other reindeer. Could the deer have transmitted the virus to humans? There is no evidence that animals, including reindeer, play an important role in the spread of SARS-CoV-2 to humans. Based on the available information, the risk of animals transmitting SARS-CoV-2 to humans is low.

Domestic cats and dogs have been repeatedly infected with SARS-CoV-2, but with few exceptions these infections are subclinical or the animals develop mild clinical disease (Bosco-Lauth et al., 2020; Patterson et al., 2020; de Morais et al., 2020; Alluwaimi et al., 2020). Respiratory and gastrointestinal symptoms have been observed in cats. Despite proximity to infected humans, no dog or cat tested positive for SARS-CoV-2 in an immunoprecipitation test. This suggests that the transmission rate of SARS-CoV-2 between humans and domestic animals is probably very low under natural conditions (van den Brand et al., 2008).

SARS-CoV-2 has been shown to replicate effectively in the digestive tract of ferrets. General clinical signs, virus replication and pathological manifestations in the lower respiratory tract of ferrets clearly indicated a high susceptibility to SARS-CoV-2 (Shi et al., 2020; Alluwaimi et al., 2020; Zhang et al., 2020a).

By experimental injection of SARS-CoV-2 was confirmed that 6 animal species (cottontail rabbits (*Sylvilagus sp*.), fox squirrels (*Sciurus niger*), Wyoming ground squirrels (*Urocitellus elegans*), black-tailed prairie dogs (*Cynomys ludovicianus*), house mice (*Mus musculus*), and racoons (*Procyon lotor*) are not susceptible to SARS-CoV-2 infection and showed no clinical signs of disease at any time during the study. But three animal species: deer mice (species *Peromyscus maniculatus),* bushy-tailed woodrats (*Neotoma cinerea*), and striped skunks (*Mephitis mephitis*) excreted infectious virus after injection. Deer mice and bush voles excreted the virus orally for <4 days, and the virus was isolated from the lungs and trachea of tested animals after 3 days with peak virus titers of 3.1log_10_ PFU/swab sample. But in the bushy-tailed forest rat, infectious virus was isolated from the lungs at a titer of 1.3 log_10_ PFU/ swab specimen, but the lungs contained 5.2 log_10_ PFU/g of virus (Bosco-Lauth et al., 2021). Striped skunks were experimentally injected with SARS-CoV-2 (strain WA1/2020WY96), and shedding of the virus orally, nasally or both was investigated. Infective virus was detected in turbinate's but not in other tissues examined, but animals did not excrete detectable virus nasally or orally before euthanasia. In general, virus titers were slightly higher in nasal samples than in oral samples, but overall peak titers in skunks were relatively low (2–2.3 log_10_ PFU/vat sample). In addition to plaque analysis, all positive animal samples were confirmed for the presence of SARS-CoV-2 by RT-PCR (East et al., 2004). Thus, without showing signs of infection, these animals excreted the virus. Rodent species have been evaluated as potential reservoir hosts or animal models for SARS-CoV-2, and the results mostly indicate that outbred species, including laboratory animals, are at best only moderately exposed. Most non-transgenic laboratory mice (*M. musculus*) are resistant to infection, but transgenic humanized mice and hamsters, including Syrian hamsters (*Mesocricetus auratus*) and dwarf hamsters (*Phodopus* sp.), are highly susceptible (East et al., 2004). It therefore remains unclear why, in projects (102 projects) investigating SARS-CoV-2, for example in Germany, mice have been used more often than other animals (89,5%), especially wild-type mice (*M. musculus*), since the start of the pandemic (Schwedhelm et al., 2021). Syrian hamsters could be more suitable than mice as a more sensitive model for investigation of SARS-CoV-2 infection (Alluwaimi et al., 2020). Dwarf hamsters have been described that became ill and died within 3 days of infection. Given that there are >1700 species of rodents worldwide, many of which live in close proximity to humans, many questions about SARS-CoV-2 in wild rodents remain unanswered. Both carnivores and wild rodents are potentially high-risk groups for SARS-CoV-2 infection. Based on the primary amino acid sequences of angiotensin-converting enzyme 2 (ACE2), transmembrane serine protease type 2 (TMPRSS2) and spike protein in different species, it was predicted that members of different taxonomic groups are susceptible to the virus (Luan et al., 2020).

*Damash J., et all.* (Damas et al., 2020) identified 410 vertebrate species with ACE2 orthologues, including representatives of all vertebrate taxonomic classes. Among them were "252 mammals, 72 birds, 65 fish, 17 reptiles and 4 amphibians". The similarity of human ACE2 to the known SARS-CoV-2 S-binding residue was investigated. In Old World primates and great apes, the Spike-binding residue 25 of ACE2 is identical to that of human ACE2. The authors suggested that "many of the 21-primate species found in China may be potential reservoirs for SARS-CoV-2. The remaining primate species were rated as high to medium risk, and only the grey mouse lemur and the Philippine tarsier were rated as low risk" (Damas et al., 2020). Similar levels of affinity to SARS-CoV-2 were obtained between different animal species based on the structural similarity of their ACE2 receptors (Wan et al., 2020).

Analysis of ACE2 domain excluded the racoons (*P. lotor*) as potential hosts for SARS-CoV-2 (Bosco-Lauth et al., 2021). Some study demonstrates that raccoon dogs (*Nyctereutes procyonoides*) (which show only subtle clinical signs) are susceptible to SARS-CoV-2 infection and can transmit the virus to direct in-contact animals. The authors found evidence of viral replication and tissue lesions in only the nasal conchae (Freuling et al., 2020).

Another sequence analysis showed that ACE2 from western spotted skunk (Spilogale gracilis) bound weakly to SARS-CoV-2 Spike. Similarly, weak binding was predicted for *Neovison vison (“American mink*”) However, this assumption is not accurate. In recent months, outbreaks of SARS-CoV-2 on commercial mink farms have been reported in Europe and recently in the USA (Bosco-Lauth et al., 2021). Some authors suggest that ruminant four-legged ungulates may serve as a reservoir for SARS-CoV-2 and that rodents cannot be excluded as possible intermediate hosts of SARS-CoV-2 (Bosco-Lauth et al., 2021; Damas et al., 2020).

##### Search for SARS-CoV-2 in birds

Through their migration, both bats and birds are able to connect distant corners of the planet. Together they can carry viruses, bacteria and parasites. Birds on a global scale are also an important host reservoir for a number of zoonotic diseases (Wille and Holmes, 2020). Therefore, it was suggested that birds could be reservoirs or carriers of SARS-CoV-2.

Birds have long been known as hosts for *Coronaviridae* members and today have been detected in 108 wild bird species (Wille and Holmes, 2020; Rahman et al., 2021) in which there's from the gamma-coronavirus (four species) and delta coronaviruses (seven species) groups dominate (Wille and Holmes, 2020). In the 1930s, the first avian coronavirus disease was described by *Schalk and Hawn* (1931) - avian infectious bronchitis (IB) (Schalk and Hawn, 1931), and *Bushnell* L.*D. and Brandly C.A.* (1933) (Bushnell, 1933) concluded that a filterable agent was its cause which confirmed by electron microscopy in 1964 *Berry* et al. as coronavirus (Berry and Chu, 1964). It was infectious bronchitis virus (IBV), which remains an economically important respiratory virus for poultry in several countries today (Najimudeen and Cork, 2020), high IB-associated losses are recorded in spite of control attempts using live attenuated vaccines. To date, SARS-CoV-2 has not been established in birds, even in experimental infection of chickens and ducks (Shi et al., 2020; Wille and Holmes, 2020). Considered is not likely to infect chickens or other poultry. The main reason for non-infection in birds consider that it is for both viruses (SARS-CoV-2 and avian viruses), have different receptors in the hosts and belong to phylogenetically different groups (Iglesias-Osores, 2020).

But, it is believed (*Michelle Wille, Edward C Holmes,* 2020) that a cross-species transmission event of coronaviruses from birds to mammals has occurred relatively recently (Wille and Holmes, 2020; Lau et al., 2018). Interestingly, the fact that PorCoV HKU15 and SpCoV HKU17 belong to the same species suggests that the interspecific transition from birds to pigs may have occurred relatively recently. BEAST (Bayesian Evolutionary Analysis Sampling Trees) analysis showed that CoV transitioned from birds to mammals about 523 years ago (Woo et al., 2012). Need to consider that the mixing of birds, pigs and other mammals in domestic and wildlife markets, as well as their close contact with humans, may create a favorable environment for the interspecific transition and subsequently risk further genetic changes to adapt to the human host, as in the case of SARS (Woo et al., 2012; Prinzinger and Schleucher, 1991). This concern may be supported by the fact that in recent years, gamma-coronaviruses and delta-coronaviruses have been found to occur not only in birds but also, for example, in pigs. Besides pigs, delta-coronaviruses have also been described in Asian leopard cats (*Prionailurus bengalensis*) and Chinese ferret badgers (*Melogale moschate*) on China markets (Dong et al., 2007): these viruses are among the first described delta-coronaviruses in mammals. The fact that these viruses have only been described in wet markets and/or exotic mammals in this one study is extremely concerning, as it highlights the propensity for these viruses to spread to mammals and the extensive under sampling of delta coronaviruses in mammals (Wille and Holmes, 2020). This also demonstrates that coronaviruses previously thought to be found only in one class of animals are now being detected in others.

## 2 Temperature factor influence on coronaviruses

### 2.1 Identification of temperature regimes and possible resistance of coronaviruses, especially of SARS-CoV-2

For all viruses, there is an optimal temperature at which they efficiently replicate in sensitive cells or storage.

There is some data on this investigation. For example, SARS-CoV-2 was reported to replicate 10-fold more efficiently at temperatures associated with the upper respiratory tract (33 °C) compared to core body temperature (37 °C) between 72- and 96-hours post-infection (Herder et al., 2021). SARS-CoV and SARS-CoV-2 exhibit strong temperature-dependent differences in replication kinetics in cell culture, "indicating an influence of host determinants during the post-initiation phases of the virus life cycle. Importantly, the significant increase in replication of SARS-CoV-2 at 33 °C likely confirms the enhanced replication in the upper respiratory tract and transmissibility of SARS-CoV-2 compared to SARS-CoV" (Herder et al., 2021). The author reported delayed activation of the innate immune system and proinflammatory pathways at 33 °C. A recent "genome-wide CRISPR screen showed that replication of SARS-CoV-2 requires partially different host factors when incubated at either 33 °C or 37 °C" (Herder et al., 2021; V'Kovski et al., 2021a; Schneider et al., 2021; P. V'Kovski et al., 2021).

Given the above, it is likely that pangolins, with their body temperature, may be reservoirs of the SARS-CoV-2 virus. Only by being exposed to suitable conditions, i.e. a human with a body temperature suitable for viral replication or another sensitive system, does the virus acquire the ability to replicate in the presence of sensitive receptors on the surface of human or animal cells. This is the first suggestion of a reservoir in which SARS-CoV-2 can survive in nature for long periods of time.

The respiratory transmission of SARS-CoV-2 is directly related to the temperature gradient in the human respiratory tract. According to some authors, it ranges from 30 to 32 °C in the nasal mucosa, rises to 32 °C in the upper trachea and reaches about 36 °C in the bronchi (Prevost et al., 2021; Lindemann et al., 2002; McFadden et al., 1985). However, the replication of coronaviruses at febrile body temperatures remains poorly characterized (Herder et al., 2021). Thus, it remains unclear whether seasonal changes in ambient temperature alter the spread of the COVID-19 epidemic (McFadden et al., 1985; Mu et al., 2021).

It can be assumed that temperature factor plays an important role in the infectivity and transmissibility of SARS-CoV-2. There are reports that the dissociation of the interaction between the viral S-protein and ACE2 receptor slows down at low temperatures (as for example at 4 °C) (Gong et al., 2022). Increased interaction of the cell receptor with the spiking protein of the virus at low temperatures leads to stronger binding of the virus to the susceptible cell (Hoffmann et al., 2021).

Some studies have confirmed that viruses, including four coronaviruses (not SARS-CoV-2: CoV-229E, Feline CoV, Porcine CoV, and Rat CoV) can stay infectious much longer in cold environments (Mu et al., 2021). However, some of the data presented in the scientific literature is controversial. For example, it was shown that the spread of SARS-CoV-2 was reduced by increasing the temperature from −13.2 to 19 °C (McFadden et al., 1985; Mu et al., 2021) and the ambient concentration of ozone (UV radiation) (Mu et al., 2021; Yao et al., 2020a). However, in the same year 2020, neither ambient temperature, relative humidity nor UV irradiation was found to be associated with transmissibility of COVID-19 (Duan et al., 2003). Some studies with virus-contaminated surfaces incubated at 20, 30 and 40 °C and sampled at different time points show that infectious SARS-CoV-2 can be recovered from non-porous surfaces at ambient temperature and humidity (20 °C and 50% RH) for at least 28 days. Increasing the temperature while maintaining the humidity resulted in a drastic reduction in the infectivity of the virus to only 24 h at 40 °C (Yao et al., 2020b).

#### 2.1.1 Possibility of long-term storage of coronaviruses

We have already performed a number of experiments on the long-term storage of porcine coronaviruses at different temperatures (Yao et al., 2020a; Duan et al., 2003; Y. Yao et al., 2020). Research has been done on investigation of the long-term effects on coronavirus, the causative agent of transmissible swine gastroenteritis (TGE), at different temperatures: + 4 °C, + 25 °C, - 13 °C, - 20 °C and thirteen temperature changes from minus 13 °C to room temperature (+18 °C to +20 °C), representing a temperature difference of 31–33 °C. Both the vaccine and the field strains of animal TGE coronavirus were found to reduce their infectivity after long-term storage, but to recover their infectivity rather rapidly (by successive in a sensitive system) when exposed to a sensitive biological system (*in vitro*).

The virus population became more homogeneous after long-term storage in terms of S-signature (plaque formation). It was observed and demonstrated that TGE coronavirus, when stored for more than two years, reduced but did not lose its infectious properties at temperatures of minus 13 °C and minus 20 °C, which were restored upon subsequent passage in sensitive biological systems in vitro. A similar observation was made when the virus was stored for 8 years at a temperature of + 4 °C without light in lyophilizes condition. In these studies, the virus population (characterized by S-feature) became more homogeneous. It can be assumed that SARS-CoV-2 also can hypothetically maintain its infectious properties at a convenient temperature, as for example by + 4 °C for a long time (e.g. 8 years) if stored without light (Iglesias-Osores, 2020) as for example in pangolin or other caves.

The most rapid decrease in pig coronavirus titer occurred at a temperature of + 25 °C within 3 days. Moreover, this decrease was observed in three research pig coronavirus strains (TGEV) in the first 0–48 h (Р>0.001) drop by 50%. But, in two strains from 48 to 72 h no further decrease in infectious activity was observed, the infectious properties of the third strain (referent) continued to decrease for up to 72 h, but were not completely lost (Р<0.001) (Klestova, 2021).This raises the possibility that the virus may also persist in the environment and stay infectious for longer periods of time, e.g. in caves.

### 2.2 Search for suitable temperature conditions for the conservation of SARS-CoV-2 in nature

Let's return to the question of previous assumptions about a possible reservoir of the virus among animals, for example among pangolins or bats. Consider their living conditions.

Interestingly, some pangolins, being nocturnal animals, hide in deep burrows (up to 7.5 m deep) or in tree hollows during the day. What are the climatic conditions in caves and tree hollows? In both cases, there is no light, which is a prerequisite for the long-term persistence of the coronavirus. Another condition is the temperature of the air in caves and burrows over many years. We did not find any information on the climatic conditions in the tree burrows, but we can assume that the temperature decreases in winter and increases in summer and fluctuates according to external conditions, as the thickness of the trees is insufficient to maintain a stable temperature for a long period of time. Therefore, it can be assumed that pangolin caves may be a place with constantly uniform climatic conditions. What are the conditions in such caves? We have not found any specific information on pangolin burrows, but after a search in the literature we have found information on climatic conditions in burrows of rodents, marmots, etc., which have been studied in relation to their possible involvement in plague occurrence.

In the marmot winter burrow (5–7 m from the surface), the air temperature does not drop below 0 °C, even during severe frost at the surface. Some authors described that in the burrows of 2.2 m or more, seasonal fluctuations in soil temperature at the level of nest chambers range from −3.5 to +2.0 °C (Сунцов, 2006). At a depth of 1.6 m in Transbaikalia, the temperature in the burrow is not below minus 8 °C during the 4–5 winter months (Некипелов, 1957). In shallower winter and summer burrows (up to 1.5 m), seasonal fluctuations are significantly greater, and the temperature remains below 0 °C for about 6 months. The minimum temperature of the soil in the winter chamber was recorded in a hole located on the northwestern slope at a depth of about 1 m, and was equal to −8.1 °C. The maximum temperature of the walls of the winter chamber, which was +7.6 °C, was recorded at a depth of 1.4 m in a hole located on the southern slope (Сунцов, 2006).

In case of the Mongolian marmot, the temperature in the burrow during winter is about + 5 °C, at a depth of 2.2 m, the temperature at the level of the nest chambers varies between - 3.5 °C and + 2 °C. In other caves at a depth of 1.5 m, the temperature is between 0 °C and +7.6 °C for 6 months. In the burrows up to 3.5 m deep, the temperature does not rise above + 8 °C (from minus 3 °C to + 8 °C) (Бибиков 1967).

We can assume that the conditions in the burrows of pangolins or other animal species can be suitable for long-term storage of the virus (provided it is introduced into the burrows by the virus carrier). Pangolins breed once a year, with the female giving birth in the nest chamber of the burrow in November-December or January-March.

The period November-December 2019 is interesting, if we continue to think about the 2020 COVID-19 pandemic when the first case indicated in human in December 2019. Aren't baby pangolins a more favorable model for the virus?

All these considerations arose after it was reported that the pangolin coronavirus was 91.02% (Zhang et al., 2020b) identical to the SARS-CoV-2 genome of infected people. However, pangolins may not be the only intermediate hosts of the pathogen. Later, the version with the virus-carrying pangolins came into question. Let us now consider the possibility that bats can be the carrier species and the possibility of SARS-CoV-2 persistence in bat caves.

Let us analyze the microclimate of caves where bats may live. The climate of caves is determined by two main factors: the influence of the temperature environment of the rocks in which the cave is located, and the influence of external factors (external climate and water flows). The air temperature inside such a cave will be stable and equal to the temperature of the walls around the cavity. However, this approach does not work for large caves. In flooded caves, zoning can be disturbed, as the heat capacity of water is about 30 times higher than the heat capacity of air. Cave water affects the climate more intensely than air. For example, in the Red Cave in the Crimea (Ukraine), the average temperature of the lower floors is +8 °C, and that of the upper floors is + 11 °C. Thus, in theory, the coronavirus can persist in bat caves for a long time. It is necessary to know exactly in which caves those bats live, namely in China, which may be carriers of the coronavirus, from which the pandemic virus has now been isolated. Do bats live near Wuhan, or even in the city?

Notably, the temperature in the far parts of the caves is equal to the average annual air temperature outside the cave only on the coastal areas of the continents. And inside the continents, the temperature in the far parts of the caves is always higher than the average annual temperature, and towards the center of the continents the temperature difference inside and outside the caves increases. For example, in the caves of Podolia, this difference is 1–2 °C, in the caves of the Urals 3–4 °C, in the caves of the Pamirs 6–7 °C. It turned out that according to the classification, all caves in Russia are warm (or temperate caves) regardless of whether they have ice or not. This also applies to artificial underground caves. Thus, we can assume that the caves in China can be also warm. Thus, it is necessary to study their temperature regime and establish how long the virus stay in the caves where bats live. This is a reflection on a possible reservoir of the virus in the environment. The location of Wuhan and its peculiarities in terms of the possible location of the natural reservoir of the coronavirus are interesting. Unfortunately, we do not have personal experience with the natural and biological features of the ecosystem of this city.

According to literary sources, the city of Wuhan consists of three historic parts. They are opposite each other on different banks of the rivers (Yangtze and Hanshui). The city center is located on a plain, while the southern part of the city is on the hills. Theoretically, there could be caves in these hills where bats live. The other part of the city is shrouded in swamps and lakes. Wuhan has a subtropical climate with monsoons and four distinct seasons. Summers and winters are warm. The average temperature in January is +3.0 °C, while the average temperature in June is +29.3 °C. Summer has up to 135 days, while spring and autumn last about 60 days. During the summer season, the rainfall reaches 1205 mm. The frost-free period is 240 days. Moreover, take into account that the city is densely populated - nearly 12 million people live in it (a lot by the number of susceptible biological objects).

Thus, the temperature conditions of the caves where the animals live are quite suitable for the long-term preservation of the infectious properties of coronaviruses (including SARS-CoV-2).

## 3 Comparing different animal's body temperature and presence of SARS-CoV-2

While many animals have a stable body temperature, others do not (Nespolo et al., 2021). This depends on many factors (including in the case of warm-blooded animals). One such factor is homeothermy (Precht et al., 1973). Homeothermy is the result of an evolutionary process in which each increase in oxygen intake led to a successive increase in metabolic rate and thus to a new dependence on favorable environmental conditions. In response to food shortages during the winter months, some inhabitants of temperate climates have developed the ability to hibernate, characterized by a fully thermally controlled drop in body temperature to values close to zero. Hibernation thus demonstrates that in homeotherms, not only is the body surface poikilothermic, but also the core temperature is more variable than is often assumed. However, unlike clinical hypothermia, natural hibernation is not a cold-induced decrease in metabolic rate, but an endogenous decrease in metabolic rate followed by a decrease in body temperature (Singer, 2007). The possible metabolic suppression factor can be pH, that is kept constant at 7.4 in hibernators by relative hypoventilation (pH-stat). In the poikilothermic body shell (alpha-stat) observed its passive shift. It is known that the normal body temperature of most mammals is 37–39 °C as a homeothermic temperature, according to other authors 36–38 °C (*Hickman* et al.*, 1984*). However, we considered the range of body temperatures of animals in which SARS-CoV-2 was detected (Table 1).

**Table 1:** Body temperatures of animal's in which SARS-CoV-2 has been detected.

Animal species	Normal core body temperature (range)	Refs. (on body temperature)	Refs. (on SARS-CoV-2 detection)
Cats *(Felis catus),* Domestic Cat *(Felidae)*	38.1 °C and 39.2 °C 38.3–39.2 °C 37.7–39.2 °C 38–39 °C 37.96–39.13 °C 36.7–38.9 °C	(Aiello, 2016) (Bashor et al., 2021) (Levy et al., 2015)	(Decaro et al., 2021; Shi et al., 2020; Garigliany et al., 2020; Bosco-Lauth et al., 2020; Patterson et al., 2020; de Morais et al., 2020; van den Brand et al., 2008; Alluwaimi et al., 2020; Q. Zhang et al., 2020)
Domestic dogs *(Canis lupus familiaris)*	38.3–39.2 °C 37.8–39.9	(Aiello, 2016; McNab, 1970; Kreissl and Neiger, 2015)	(Decaro et al., 2021; Shi et al., 2020; Sit et al., 2020; Bosco-Lauth et al., 2020; Patterson et al., 2020; de Morais et al., 2020)
Minks *(Neovison vison, Mustela lutreola)*	36–38 °C 38.6–39.7 °C 38.4–40.3 °C	(von Jr. Cleveland et al., 1984) (Harjunpaa and Rouvinen-Watt, 2004)	(Boklund et al., 2021; Shriner et al., 2021; Oreshkova et al., 2020)
Otter *(subfamily Lutrinae*)	38–38.52 °C 37.3–39.04 °C 35.9–40.4 °C	(Body temperature. Eurasian Otters. 04 Jul 2022; Kruuk, 1997) (Costa, 1982)	(SARS-COV-2 in animals – situation report 12 30/04/2022, SARS-COV-2 in animals – situation report 1 31/05/2021; Confirmation of COVID-19 in Otters at an Aquarium in Georgia 2021)
Pet ferret *(Mustela putorius furo)*	37.8–40 °C	(Schoemaker, 2008)	(SARS-COV-2 in animals – situation report 12 30/04/2022, SARS-COV-2 in animals – situation report 1 31/05/2021; Shi et al., 2020; van den Brand et al., 2008; Alluwaimi et al., 2020)
Lion *(Familie: Felidae*, genus *Felis)*	36.81–37.95 °C (37.3–37.5 in winter)	(Friend et al., 2020)	(SARS-COV-2 in animals – situation report 12 30/04/2022, SARS-COV-2 in animals – situation report 1 31/05/2021; Mishra et al., 2021; Koeppel et al., 2022)
Tiger *(Panthera tigris)*	37.8–39,4 °C	(McNab, 2000)	(SARS-COV-2 in animals – situation report 12 30/04/2022, SARS-COV-2 in animals – situation report 1 31/05/2021; Mitchell et al., 2021; Grome et al., 2022)
Puma *(Felis concolor)*	37.6 ºC	(McNab, 2000; AnAge: The Animal Ageing and Longevity Database 26 January 2022)	(SARS-COV-2 in animals – situation report 12 30/04/2022, SARS-COV-2 in animals – situation report 1 31/05/2021; Koeppel et al., 2022)
Snow leopard *(Panthera uncia)*	36.9–39.3 °C	(Esson et al., 2019)	(SARS-COV-2 in animals – situation report 12 30/04/2022, SARS-COV-2 in animals – situation report 1 31/05/2021; Wang et al., 2022)
Gorilla *(Gorilla gorilla)*	35.8–35.7 °C	(Brown and Finnegan, 2007)	(SARS-COV-2 in animals – situation report 12 30/04/2022, SARS-COV-2 in animals – situation report 1 31/05/2021; Erste Gorillas mit dem Coronavirus infiziert, in Scinexx 2022)
White-tailed deer *(Odocoileus virginianus)*	38.5–40.6 °C	(Nunez et al., 2020)	(Chandler et al., 2021; Questions and Answers: Results of Study on SARS-CoV-2 in White-Tailed Deer 2021)
Fishing cat *(Prionailurus viverrinus)*	37.7–39.1 °C	(Sunquist, 2002)	(SARS-COV-2 in animals – situation report 12 30/04/2022, SARS-COV-2 in animals – situation report 1 31/05/2021; Confirmation of COVID-19 in a Binturong and a Fishing Cat at an Illinois Zoo 2021)
Binturong (*Arctictis binturong)*	36.7–37.4 °C	(Moresco and Larsen, 2003)	(SARS-COV-2 in animals – situation report 12 30/04/2022; Confirmation of COVID-19 in a Binturong and a Fishing Cat at an Illinois Zoo 2021)
Spotted hyena *(Crocuta crocuta*)	36.1 °C	(McNab, 2000; Flies et al., 2015)	(SARS-COV-2 in animals – situation report 12 30/04/2022; East et al., 2004)
Manatees *Trichechus manatus latirostris (genus Trichechu*s)	35.0–35.8 ºC	(Martony et al., 2020)	(SARS-COV-2 in animals – situation report 12 30/04/2022)
Eurasian lynx *(Lynx lynx)*	36–38ºC	(C.J., 2004)	(SARS-COV-2 in animals – situation report 12 30/04/2022, SARS-COV-2 in animals – situation report 1 31/05/2021)
Canadian lynx *(Lynx canadensis*)	36.39–37.8 °С (30.67–41.085 ºC)	(Sunquist, 2002) (Solomonov, 2016)	(SARS-COV-2 in animals – situation report 12 30/04/2022, SARS-COV-2 in animals – situation report 1 31/05/2021; Lynx TESTs POSITIVE FOR SARS-CoV-2 2021)
Hippopotamus *(Hippopotamus amphibius)*	36.7–37.8 ºC	(Wright, 2015)	(SARS-COV-2 in animals – situation report 12 30/04/2022; Hayashi, 2022)
Hamster *(subfamily Cricetinae)*	34.92 °C 36.1–38.9 °C	(Chayama et al., 2016) (Haugg et al., 2021)	(SARS-COV-2 in animals – situation report 12 30/04/2022; Bosco-Lauth et al., 2020; Alluwaimi et al., 2020)
Mule deer *(Odocoileus hemionus)*	37.5–39.7 °C	(Sargeant, 1994)	(SARS-COV-2 in animals – situation report 12 30/04/2022; Mallapaty, 2022)
Skunks *(family Mephitidae)*	35–36 °C	(Skunk First Aid. 03/20/14)	(Bosco-Lauth et al., 2021)
Giant anteater *(Myrmecophaga tridactyla)*	32.7–33⁰C	(Castagnino, 2021, Constança de Sampaio; Moura et al., 2021)	(SARS-COV-2 in animals – situation report 12 30/04/2022; Castagnino, 2021)
Coati (coatimundi), *(Nasua narica)* South American coati *(Nasua nasua)*	38–39 °C 36.4ºC	(AnAge: The Animal Ageing and Longevity Database 26 January 2022)	(SARS-COV-2 in animals – situation report 12 30/04/2022; Confirmation of COVID-19 in a Coatimundi at an Illinois Zoo 2021)
Pangoline *(order Pholidota, Family Manidae*) (PALAWAN PANGOLIN (Manis culionensis) CONSERVATION STRATEGY 2018–2043 2020) (*Schlitter 2005*)	30.6–34.2 °C	(Yu et al., 2021)	(Temmam et al., 2022; Liu et al., 2020; Wahba et al., 2020; Wege et al., 1982)
Black-tailed marmoset *(Mico melanurus)*	35.4–39.1 °C	(Morrison, 1962)	(SARS-COV-2 in animals – situation report 12 30/04/2022; Pereira et al., 2022)
Bats *(order Chiroptera)*	36.9–41.3 °C	(O'Shea et al., 2014)	(Shi et al., 2020; Delaune et al., 2021)

It is noticeable that in some animals there are quite large ranges of fluctuations in body temperature. It is known that the body temperature of animals depends on various circumstances. For example, on the activity of the animals, on changes depending on the phases of the day, on the seasons, etc. Since the diets and natural habitats of all the above animals (diagnosed with SARS-CoV-2) are different, we can only assume that the preservation of the virus in nature (in animals) can depends on the presence of a specific virus receptor and the body temperature of the animals, where the virus can persist or multiply for a long time.

Our data review revealed that some animals have a low body temperature, lower than 37–39 °C. These include animals such as the giant anteater (32.7–33 °C), the pangolin (30.6–34.2 °C), the hamster (34.92 °C), manatees (35–35.8 °C), the black-tailed marmoset (35.4 °C), the gorilla (35.8–35.7 °C), the otter (35.9 °C), the mink (36 °C), the skunk (35–36 °C) and the spotted hyena (36.1 °C). Interestingly, there is also a strong fluctuation of the body temperature over the year in some species, as illustrated by the Canadian lynx (Table 2) (Solomonov, 2016).

**Table 2:** Average daily body temperature of a Lynx during the year (in °C ).

Month		Male 1	Male 2
October	Min-Max	34.24–39.099	33.29–39.5
November	Min-Max	31.62–38.97	31.29–39.47
December	Min-Max	31.24–39.66	33.67–39.34
January	Min-Max	31.24–38.79	32.29–38.72
February	Min-Max	31.93–38.97	32.11–38.84
March	Min-Max	33.05–39.59	32.61–38.47
April	Min-Max	33.93–40.59	32.98–38.47
May	Min-Max	34.98–41.03	32.79–39.90
June	Min-Max	34.55–40.34	32.86–41.085
July	Min-Max	33.92–40.59	32.60–39.34
August	Min-Max	34.36–40.16	30.67–39.28
September	Min-Max	34.67–39.84	34.04–38.90

As illustrated by the data in Table 2, there is a remarkable variation in the body temperature of Lynx throughout the year. Looking at the lower values, they are in the range of 30.67–34.98 °C. This temperature is better for SARS-CoV-2 replication and interaction with ACE2 (McFadden et al., 1985; Hoffmann et al., 2021; Higgs et al., 2014) than higher temperatures.

As another example, consider giant anteaters. They have one of the lowest body temperatures of all placental mammals, as some authors write (but we see that the lowest may be in the lynx), around 32.7–33 °C, and they have a low capacity for physiological thermoregulation. Therefore, they rely on behavioral adaptations for thermoregulation. The body temperature of giant anteaters depends on the ambient temperature. Giant anteaters are more sensitive to cold than to heat, probably due to low body heat production (Castagnino, 2021).

Other animals, such as the Eurasian otter, show similar fluctuations in temperature. The Eurasian otter (on land and in water) has an average body temperature of 38.1 °C. However, the lowest measured body temperature was 35.9 °C and the highest was 40.4 °C, a difference of 4.5 °C (Kruuk, 1997). Body temperature was high when an otter entered the water, significantly higher than the average. This observation has also been made for other species such as the muskrat (*Ondatra zibethicus*) (Kruuk, 1997; Costa, 1982). This may be due to increased activity on land before diving into the water. During swimming, their body temperature decreases. In this case, a low body temperature gives animals a greater chance of being infected with SARS-CoV-2.

In ongoing experiments, especially with SARS-CoV-2, body temperature fluctuations in other animals must also be considered. It is also possible to consider the immediate reaction of animals to an abrupt change from LP (long photoperiod) to SP (short photoperiod). For example, in hamsters (*Phodopus sungorus*) body temperature decreased during SP activity, indicating profound physiological changes at the very beginning of the adaptation phase. This emphasizes the importance of careful control of photoperiod regimes in experimental animals and suggests more focus on the initial metabolic adaptation profiles of SP by conducting experiments examining coronavirus infection (Haugg et al., 2021).

Some pangolins live in open areas, savannas, and tropical forests (both terrestrial and arboreal), feed on ants and love to swim. In addition to ants, pangolins eat other insects and their larvae, as well as snails, worms, snakes, berries, roots. They lead a nocturnal lifestyle. An important factor for us is their body temperature, which is 31.7–34.2 °C. There is information that the body temperature of these animals varies greatly depending on the ambient temperature. For exemple, the body temperatures: in Chinese pangolins (*Manis pentadacyla*) was 33.2 ± 0.95 °C (30.6–34.2 °C, *n* = 3) and in Sunda pangolins (*M. javanica*) was 32.8 ± 0.48 °C (31.7–34.2 °C, *n* = 3). The daily variation in body temperature was 1.0–2.9 °C in Chinese pangolins and 1.2–1.9 °C in Sunda pangolins (Yu et al., 2021). *Heath, M.E. and H.T. Hammel* (1987) collected continuous body temperature data on Chinese pangolins and the results show an obvious body temperature fluctuation rhythm (Heath and Hammel, 1986). Body temperatures were lower during the day than at night, with the highest temperature around midnight and the lowest around noon (Yu et al., 2021; Heath and Hammel, 1986). The body temperature of Sunda pangolins tended to rise at night too, with the highest body temperatures occurring mostly at night (Yu et al., 2021). Analysis of temperature regimes in pangolins was carried out to find out if their body temperature is suitable for virus SARS-CoV-2 replication or not. Previously, it could be considered that SARS-CoV-2 is 'persistent' only under these conditions (at the temperature is below 36–37 °C) and does not replicate in these animals, as we also assumed in the case of pangolins. It was also thought that these animals could be 'carriers' of SARS-CoV-2 without clinical signs under these conditions. Since earlier, when studying porcine coronavirus TSE, it was found that the optimal temperature is 36.8–37 °C. This was the view before researchers learned about the optimal temperature for SARS-CoV-2 replication. But now is links that the temperature of 33 °C (Herder et al., 2021) is better for SARS-CoV-2 replication (higher titres) than higher temperatures, for example than at 37 °C. Let us return to the situation with birds, where it was not possible to detect SARAS-CoV-2 either in the wild or under experimental conditions. But could it be because the body temperature of birds (Prinzinger and Schleucher, 1991) is higher than that of mammals (usually this range is 39–43 °C)? And there is also evidence that it is 1.87 °C higher at rest and 2.43 °C higher in the active phase (Prinzinger and Schleucher, 1991).

We can speculate that SARS-CoV-2 and related respiratory beta-coronaviruses can replicate and spread efficiently in animals with low body temperatures (around 33 °C). Low temperatures enhance the interaction between SARS-CoV-2 Spike and ACE2, and ACE2 binding to the cell surface of SARS-CoV-2 Spike was higher at low temperature (4 °C) compared to 37 °C (Wan et al., 2020). It follows that low temperatures promote Spike-ACE2 interactions regardless of Spike trimer stability and the resulting mutations (Wan et al., 2020). The authors observed temperature-dependent interaction between SARS-CoV-2 Spike protein and ACE2 (Wan et al., 2020). Therefore, the above-mentioned animals in which SARS-CoV-2 has been detected, having a low body temperature between 30 and 37 °C and expressing a suitable ACE2 receptor, could potentially represent reservoirs of the virus.

The above analysis thus provides an indication that the circulation of SARS-CoV-2 in the nature is becoming more widespread and that new concepts and strategies for preventive measures to protect human and animal health should be developed.

## 4 Conclusion

The wide distribution of SARS-CoV-2 in animals in different regions of the world has been demonstrated that regional spreading of coronaviruses requires further careful monitoring. By comparative analysis it is found that many of warm-blooded animals can be suitable hosts for the replication of SARS-CoV-2 and transmit the virus to other susceptible animals or, perhaps, to humans. It was considered one possible environmental factor (temperature) and related ways in which the virus can spread and circulate in wildlife. One important factor determining the carriage or successful replication of the virus can be the body temperature of animals and its fluctuations. It was stressed that many wild animals have a specific receptor and optimal temperature for virus replication. Once in the wild, the virus has all conditions and chances to circulate and transmit to other susceptible organisms for a long time. It is founded that humanity is likely to coexist for a long time with this emergent pathogen, as it is already circulating not only among the human population, but also among fauna and wild animals in different regions of the planet. Thus, the range of the virus may expand, and there is more than enough material and conditions for its evolution.

## Acknowledgments

The author would also like to thank Prof., Dr. Daniel Sauter for comments and fruitful discussions, and the Institute for Medical Virology and Epidemiology of Viral Diseases, Tübingen, for support.

## Funding agency

This work was supported by the Research@Tübingen Fellowship of The University of Tübingen and the DAAD.

## Declaration of Competing Interest

The authors declare that they have no known competing financial interests or personal relationships that could have appeared to influence the work reported in this paper.

## Data Availability

Data will be made available on request.
